# Simultaneous Inhibition of BCR-ABL1 Tyrosine Kinase and PAK1/2 Serine/Threonine Kinase Exerts Synergistic Effect against Chronic Myeloid Leukemia Cells

**DOI:** 10.3390/cancers11101544

**Published:** 2019-10-12

**Authors:** Sylwia Flis, Ewelina Bratek, Tomasz Chojnacki, Marlena Piskorek, Tomasz Skorski

**Affiliations:** 1 Department of Pharmacology, National Medicines Institute, Chełmska 30/34, 00-725 Warsaw, Poland; ebratek@imdik.pan.pl (E.B.); m.piskorek@nil.gov.pl (M.P.); 2 Department of Hematology, Military Institute of Medicine, Szaserów 128, 04-141 Warsaw, Poland; tchojnacki@wim.mil.pl; 3 Temple University School of Medicine, Sol Sherry Thrombosis Research Center and FELS Institute for Cancer Research & Molecular Biology, Philadelphia, PA 19140, USA

**Keywords:** chronic myeloid leukemia (CML), imatinib (STI571), PAK inhibitors, PAK1/2, synergy, apoptosis

## Abstract

Tyrosine kinase inhibitors (TKIs) revolutionized the treatment of chronic myeloid leukemia in the chronic phase (CML-CP). However, it is unlikely that they can completely “cure” the disease. This might be because some subpopulations of CML-CP cells such as stem and progenitor cells are resistant to chemotherapy, even to the new generation of TKIs. Therefore, it is important to look for new methods of treatment to improve therapeutic outcomes. Previously, we have shown that class I p21-activated serine/threonine kinases (PAKs) remained active in TKI-naive and TKI-treated CML-CP leukemia stem and early progenitor cells. In this study, we aimed to determine if simultaneous inhibition of BCR-ABL1 oncogenic tyrosine kinase and PAK1/2 serine/threonine kinase exert better anti-CML effect than that of individual treatments. PAK1 was inhibited by small-molecule inhibitor IPA-3 (p21-activated kinase inhibitor III), PAK2 was downregulated by specific short hairpin RNA (shRNA), and BCR-ABL1 tyrosine kinase was inhibited by imatinib (IM). The studies were conducted by using (i) primary CML-CP stem/early progenitor cells and normal hematopoietic counterparts isolated from the bone marrow of newly diagnosed patients with CML-CP and from healthy donors, respectively, (ii) CML-blast phase cell lines (K562 and KCL-22), and (iii) from *BCR-ABL1*-transformed 32Dcl3 cell line. Herein, we show that inhibition of the activity of PAK1 and/or PAK2 enhanced the effect of IM against CML cells without affecting the normal cells. We observed that the combined use of IM with IPA-3 increased the inhibition of growth and apoptosis of leukemia cells. To evaluate the type of interaction between the two drugs, we performed median effect analysis. According to our results, the type and strength of drug interaction depend on the concentration of the drugs tested. Generally, combination of IM with IPA-3 at the 50% of the cell kill level (EC50) generated synergistic effect. Based on our results, we hypothesize that IM, a BCR-ABL1 tyrosine kinase inhibitor, combined with a PAK1/2 inhibitor facilitates eradication of CML-CP cells.

## 1. Introduction

Chronic myeloid leukemia (CML) is a clonal myeloproliferative disorder, which results from t(9;22)(q34;q11) reciprocal translocation between chromosomes leading to the formation of an oncogenic *BCR-ABL1* gene fusion. The protein product of the *BCR-ABL1* gene is characterized by constitutive tyrosine kinase activity and its activation is responsible for the deregulation of different signaling pathways pivotal for the proper functioning of hematopoietic stem cells (HSCs) [1]. Chronic myeloid leukemia in the chronic phase (CML-CP) is a leukemia stem cell (LSC)-derived disease, but the deregulation of LSC-derived leukemia progenitor cells (LPCs) leads to the manifestation of the disease [2]. CML-CP may progress to more advanced and difficult to treat phases such as accelerated phase (CML-AP) and very aggressive blast phase (CML-BP) [3]. The majority of patients with CML-CP are treated with first- or second-generation tyrosine kinase inhibitors (TKIs), which induce complete cytogenetic response (CCR) or complete molecular response (CMR) in 60–70% and only 8% of the cases, respectively [4,5]. However, complete “cure” of patients with CML, even those responding positively to treatment, using TKIs is unlikely because CML-CP LSCs are not sensitive even to second- and third-generation TKIs [6,7]. In concordance, discontinuation of TKI treatment in patients with CCR/CMR results in a relapse of the disease in the majority of cases [8,9,10].

Furthermore, 40–90% of the patients with CML express TKI-resistant BCR-ABL1 kinase mutant gene and express other genetic aberrations that frequently appear as a result of genomic instability. Such a phenomenon of acquired resistance may concern about 15–25% of patients initially responding positively to imatinib (IM) [3,11]. Second-generation TKIs (e.g., dasatinib and nilotinib) and third-generation TKIs (e.g., ponatinib) exert anti-CML effect in 40–50% of the patients who fail to respond to IM [12,13]. Unfortunately, resistance to second- and third-generation TKIs emerged due to new and/or compound BCR-ABL1 kinase mutations [14], which are associated with inferior response [15]. Altogether, CML cells, especially LSC and LPC cells, are elusive targets [16,17], and better treatment modalities are necessary to improve therapeutic outcome and to achieve cure [18].

Our reports [19,20,21,22,23], and that of others [24,25,26,27,28,29,30,31], indicate that member(s) of class Ia phosphatidylinositol 3 kinases (PI3K Ia) family and small GTP-binding protein Rac2 play a crucial role in the survival and proliferation of CML cells treated, or untreated, with TKI. Moreover, we reported that TKIs did not decrease the activity of PI3K Ia → Rac2 → p21-activated protein kinase (PAK) pathway in LSCs and LPCs in the presence of growth factors [32,33,34,35].

The family of PAK serine/threonine kinases consists of two groups: PAK1–3 and PAK4–6. Both groups share a significant level of homology but differ in the mechanisms of activation [36]. In this study, we aimed to evaluate whether blocking PAK1 and/or PAK2 activity increased the anti-CML effect of IM.

## 2. Results

### 2.1. Effects of Combination Treatment of IM with IPA-3 against CML-BP Cell Lines

**Figure 1:**
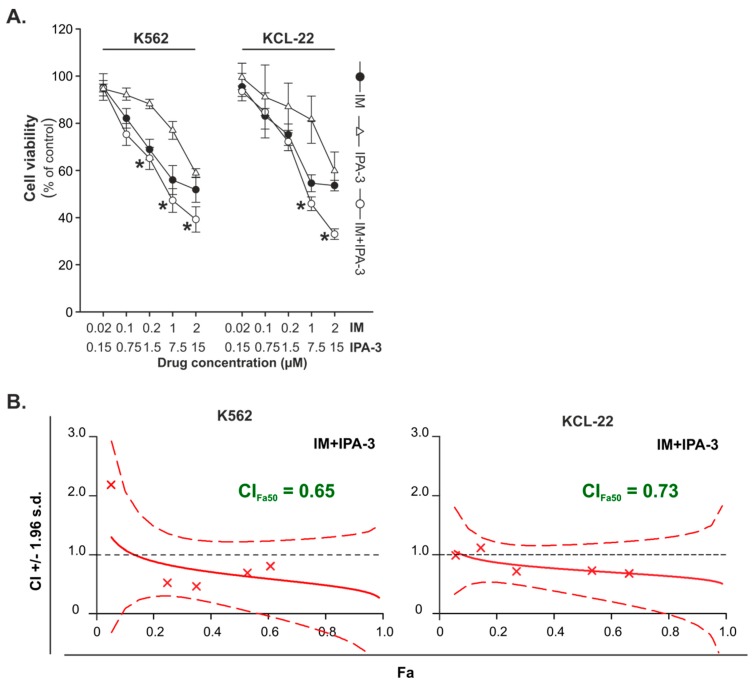
Effect of imatinib (IM) and IPA-3 on cell viability of chronic myeloid leukemia in the blast phase (CML-BP) cell lines. (**A**) The cells were treated for 24 h with the indicated doses of IM and IPA-3 individually and in combination followed by 3-(4, 5-dimethylthiazol-2-yl)-2,5-diphenyltetrazolium bromine (MTT) assay to test cell viability. Each point represents the mean ± standard deviation (SD) (n ≥ 4), * *p* < 0.05 in comparison with imatinib. (**B**) Combination index (CI) values with a 95% confidence interval at all effective levels indicate the nature of the interactions (CI = 1 = additivity, CI < 1 = synergy, CI > 1 = antagonism). Fa is the fraction of cells affected by combination treatment.

IPA-3 is a highly selective small-molecule inhibitor of PAK1 kinase [37]. The effects of IM and IPA-3 were examined on K562 and KCL-22 cell lines derived from patients with CML-BP. The cells were treated with IM in the concentration range of 0.02–2 μM and IPA-3 in the range of 0.15–15 μM. Both IM and IPA-3 were used alone or in combination. The results of the cell viability assay showed that IM and IPA-3 were more potent against K562 and KCL-22 than that of IM tested alone (Figure 1A). Analysis of the type of drug interactions revealed that the combination of IM and IPA-3 produced synergistic effect at the 50% growth inhibition level (Fa = 0.50) in K562 and KCL-22 cells (Figure 1B). Additionally, the inhibition of BCR-ABL1 kinase by evaluation the level of phosphorylated Crkl at Tyr207, the specific substrate, and biomarker for BCR-ABL1 activity, was confirmed using Western blot analysis (Figure S1).

### 2.2. Cell Cycle Perturbations and Induction of Apoptosis by IM with/without IPA-3

**Figure 2:**
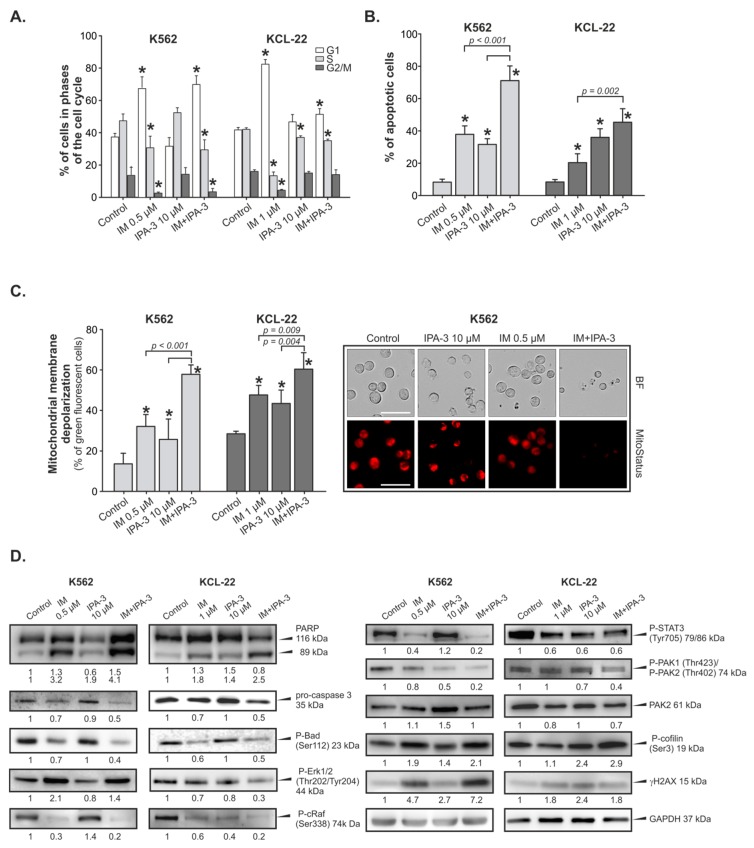
Effect of imatinib (IM), IPA-3, and their combination on cellular processes in K562 and KCL-22 cells. (**A**) Changes in the cell cycle distribution of K562 and KCL-22 cells after treatment with the indicated inhibitors. Each bar represents the mean ± SD. (*n* ≥ 4). A significant difference compared with the control group at *p* ≤ 0.003 is indicated by an asterisk (*). (**B**) Detection of apoptosis by using the Annexin V-fluorescein isothiocyanate (FITC)/propidium iodide (PI) staining. Data are expressed as the mean ± SD (*n* ≥ 5); * *p* ≤ 0.001 in comparison to control cells. (**C**) Changes in the mitochondrial membrane potential (MMP). Left panel: Depolarization of mitochondrial membrane measured by the green fluorescence of JC-1 stained cells. Data were expressed as mean  ±  SD (n ≥ 3); * *p* ≤ 0.03 in comparison to control cells. Right panel: Representative microphotographs present MitoStatus Red stained K562 cells treated with IM, IPA-3, and their combination for 24 h. A decrease in red fluorescence intensity reflects loss of MMP visualized under the fluorescent microscope (ZOE Fluorescent Cell Imager, Bio-Rad, Hercules, CA, USA), magnification 40×, scale bar 49 µm. (**D**) Western blots analysis of the indicated proteins. Glyceraldehyde 3-phosphate dehydrogenase (GAPDH) was used as a loading control. The changes in the protein levels were estimated by quantification of band intensities with the QuantiScan ver.3.0 (Biosoft, UK) densitometry analysis software. The protein levels were normalized to the signal of GAPDH (housekeeping protein). To calculate the normalized signal of each experimental target band, the signal intensities of each experimental target band were divided by the normalization factor. Normalization factor was determined by dividing the observed signal value for the GAPDH in each analyzed lane by the highest observed housekeeping protein signal on the blot. The results are presented as the arbitrary units (A.U.) calculated in comparison to control band. Abbreviations: PARP, Poly (ADP-ribose) polymerase; Bad, BCL2 associated agonist of cell death; cRaf, RAF proto-oncogene serine/threonine-protein kinase; STAT3, signal transducer and activator of transcription 3; Erk1/2, extracellular signal-regulated 1/2 kinase; γH2AX, phosphorylated form of H2A histone family member X.

To clarify the mechanism responsible for the inhibition of cells survival after IM, IPA-3 or their combination treatment, the distribution of cell cycle in K562 and KCL-22 cells was evaluated. Analysis of cellular DNA content showed that the tested compounds modified the progression of the cell cycle compared to untreated cells in both K562 and KCL-22 cells (Figure 2A). However, both cell lines demonstrated a similar response. In the K562 cells, 0.5 µM IM consistently increased the number of cells in G1 phase from ~40% to ~70% as compared to the control cells, whereas 10 µM IPA-3 had no effect on the progression of cell cycle. In the case of KCL-22 cells, IM at 1 µM concentration also significantly increased the percentage cells in G1 phase from ~40% to ~80% as compared to the control cells. A similar trend, but not statistically significant, was observed for 10 µM concentration of IPA-3. Both K562 and KCL-22 cells treated with combination of IM and IPA-3 arrested the cells in G1 phase. However, in the case of K562 cells, the strength of observed effect was the same as for cells treated only with IM. In the case of KCL-22 cells, combination treatment slightly arrested the cells in G1 phase with a concomitant reduction in the percentage of cells in S phase (Figure 2A). In addition, flow cytometric analysis of cell cycle revealed that IM and IPA-3 induced the accumulation of cells in the sub-G1 area. This effect was augmented when IM and IPA-3 were combined. In the case of K562 and KCL-22 cells, IM applied alone induced apoptosis in ~40% and 20% of the cells, respectively, whereas IPA-3 induced apoptosis in ~30% of the cells in both types of cell lines. After incubation with combination of IM and IPA-3, the percentage of apoptotic cells increased to ~70% and ~40% respectively in K562 and KCL-22 cells (Figure 2B). Changes in the mitochondrial membrane potential (MMP) are correlated to early events in the apoptotic signaling pathway. In this study, we explored the effect of IM, IPA-3, and their combination on MMP. The flow cytometric analysis revealed that the percentage of cells with depolarized mitochondrial membrane gradually increased after treatment with drugs individually. Combination treatment resulted in a greater loss of MMP in both evaluated cell lines than that of individual treatments (Figure 2C). In both cell lines, the combination treatment led to a loss of MMP in ~60% of the cell population, whereas cells treated with IM alone showed a loss of MMP in ~30% and 47% of the cell population of K562 and KCL-22 cell lines, respectively.

PAKs play an important role in the downregulation of several important proapoptotic pathways. It is known that PAK1 protects cells from intrinsic apoptotic signals via a PAK—cRaf—BAD pathway and can directly regulate the activation of the mitogen-activated protein (MAP) kinase pathway [38]. However, cRaf kinase is involved in cell proliferation through phosphorylation of mitogen-activated protein kinase (MEK) and ERK1/2 kinases. Therefore, we assumed that the inhibition of PAK1 by IPA-3 should result in the induction of apoptosis with a concomitant decrease in the activity of proteins responsible for cell proliferation (cRaf, ERK1/2) and cell signaling (STAT3). STAT3 protein was chosen because of its role as one of the downstream effectors of signaling pathways activated by BCR-ABL1 kinase leading to the transformation of CML, as well as results of the studies indicating that downregulation of STAT3 phosphorylation correlates with the induction of apoptosis of CML cells especially those that are resistant to TKIs. [39]. Therefore, Western blotting was used to detect the changes in the level of proteins responsible for the activation of apoptosis and regulation of intracellular signaling pathways. As shown in Figure 2D, incubation of cells with a combination of IM and IPA-3 resulted in a decreasing level of BAD protein phosphorylated at Ser 112 and pro-caspase 3 enzyme indicating the induction of apoptosis. Moreover, proteolytic cleavage of poly-(ADP-ribose) polymerase (PARP) protein was detected as the indirect indicator of caspase 3 activity. Changes in p-ERK1/2, p-cRaf (Ser338), and p-STAT3 (Tyr705) kinases were also observed. However, the observed effect was dependent on the evaluated cell line. According to our results, the combination of IM and IPA-3 notably reduced the level of phosphorylated cRaf protein at Ser338, whereas the reduction of STAT3 at Tyr705 was rather the effect of inhibition of BCR-ABL1 activity resulting from the action of IM (Figure 2D).

### 2.3. Response of Primary CML-CP and CML-BP Cells to IM with/without IPA-3

**Figure 3:**
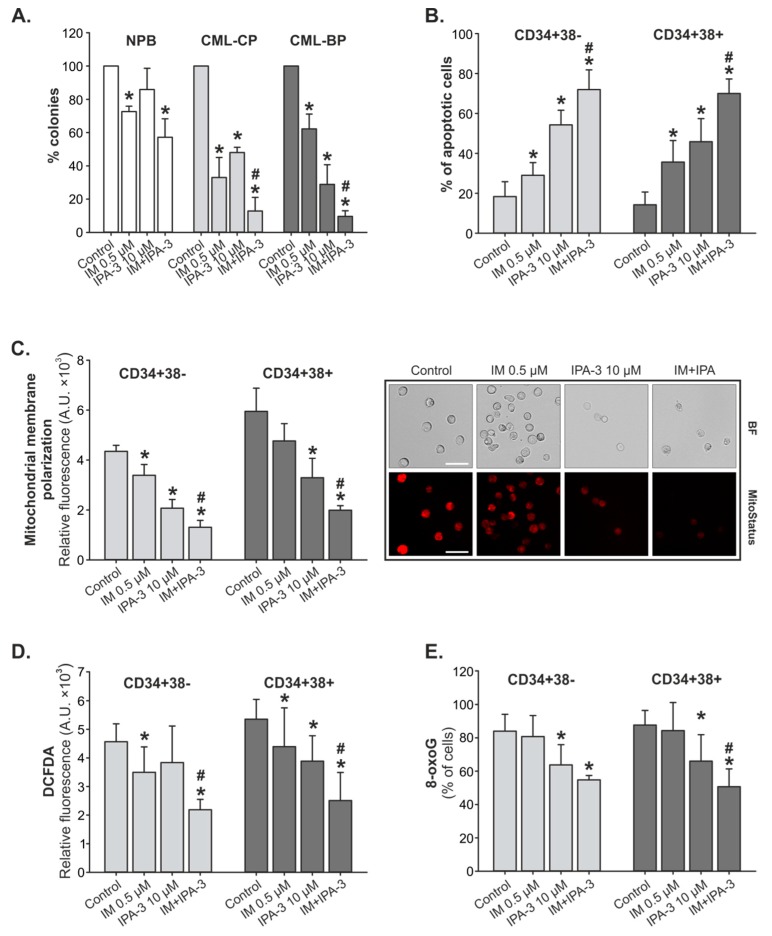
Effect of combination of IM and IPA-3 on primary chronic myeloid leukemia (CML) cells. (**A**) Clonogenic assay. Lin¯CD34^+^ primary cells obtained from healthy donors (NPB, *n* = 3) and from patients with chronic myeloid leukemia in the chronic phase (CML-CP) (*n* ≥ 5) and CML-BP (*n* = 3) were incubated with IM, IPA-3, and their combination for 72 h in the presence of growth factors. Mean percentage of colonies ± SD from triplicates, * *p* ≤ 0.01 in comparison to control (**C**), and # *p* ≤ 0.01 in comparison to IM using one-way analysis of variance Student–Newman–Keuls Method. (**B**) Apoptosis detection. Mean intensity of annexin V-fluorescein isothiocyanate (FITC)/propidium iodide (PI) staining ± SD (*n* = 8). * *p* ≤ 0.004 in comparison to (**C**) and # *p* ≤ 0.001 in comparison to IM. (**C**) Mitochondrial membrane potential. Mean fluorescence intensity (MFI) of MitoStatus Red  ±  SD (*n* = 3), * *p* < 0.008 in comparison to (**C**), # *p* < 0.003 in comparison to IM. Representative microphotographs present Lin¯CD34^+^ primary cells obtained from patients with CML-CP were treated with IM, IPA-3, and their combination for 24 h. A decrease in red fluorescence intensity reflects loss of mitochondrial membrane potential visualized under a fluorescent microscope ZOE Fluorescent Cell Imager, magnification 70×, scale bar 28 µm. (**D**) Intracellular level of reactive oxygen species. Mean 2’,9’-dichlorodihydrofluorescein-diacetate (DCFDA) staining  ±  SD (*n* ≥ 5), * *p* < 0.05 in comparison to (**C**). (**E**) DNA damage. Mean 8-oxoguanine (8-oxoG) fluorescence  ± SD (*n* ≥ 5), * *p* < 0.05 in comparison to (**C**), **#** *p* < 0.02 in comparison to IM.

Lin¯CD34^+^ primary CML-CP cells, as well as CML-BP cells, were sensitive to IM and IPA-3. Cells from patients with CML-CP were more sensitive to IM and were less responsive to IPA-3 in comparison to cells derived from patients with CML-BP. However, in both CML-CP and CML-BP samples, the combination treatment produced a decrease in the colony formation as compared to individual treatments (Figure 3A). The inhibition of colony formation of the cells derived from healthy donors, treated with a combination of drugs, was four times lower than that of cells derived from patients with CML.

Next, we detected cellular apoptosis. According to our results, the proportion of apoptotic cells increased from ~10% (control) to ~66% and ~75% for respectively CD34^+^38^−^ (LSC-enriched) and CD34^+^38^+^ (LPC-enriched) cells treated with the combination treatment. The percentage of apoptotic cells treated with IM or IPA-3 individually was significantly lower in comparison to combination treatment (Figure 3B). This correlates with the values of MMP. Cells with an increasing level of apoptosis characterized by a concomitant decrease in the MMP. As shown in Figure 3C, MMP was approximately three times lower in cells treated with combination treatment (MFI_CD34+38−_ = 1305.4 ± 276.6, MFI_CD34+38+_ = 1990.8 ± 180.0) than that of control cells (MFI_CD34+38−_ = 4348.5 ± 240.8, MFI_CD34+38+_ = 5950.7 ± 928.7) and ~2.5 times lower than that of IM (MFI_CD34+38−_ = 3389.9 ± 430.8, MFI_CD34+38+_ = 4765.1 ± 695.8) applied singly.

In this study, we performed an analysis of reactive oxygen species (ROS) using the redox-sensitive fluorochrome 2’,9’-dichlorodihydrofluorescein-diacetate (DCFDA) to detect DNA damage. According to our results, CD34^+^38^+^ subpopulation of cells was more sensitive to the combination treatment. Combination treatment downregulated the level of ROS by approximately twofold in comparison to IM applied alone (Figure 3D). A similar trend was observed during the assessment of 8-oxoguanine (8-oxoG) as the indicator of the oxidative DNA damage induced by ROS. The reduction in ROS in CD34^+^38^−^ and CD34^+^38^+^ cells was accompanied by a ~30% depletion in the accumulation of 8-oxoG (Figure 3E).

### 2.4. Response of PAK2 Silenced 32Dcl^BCR-ABL1^ Cells to IM

**Figure 4:**
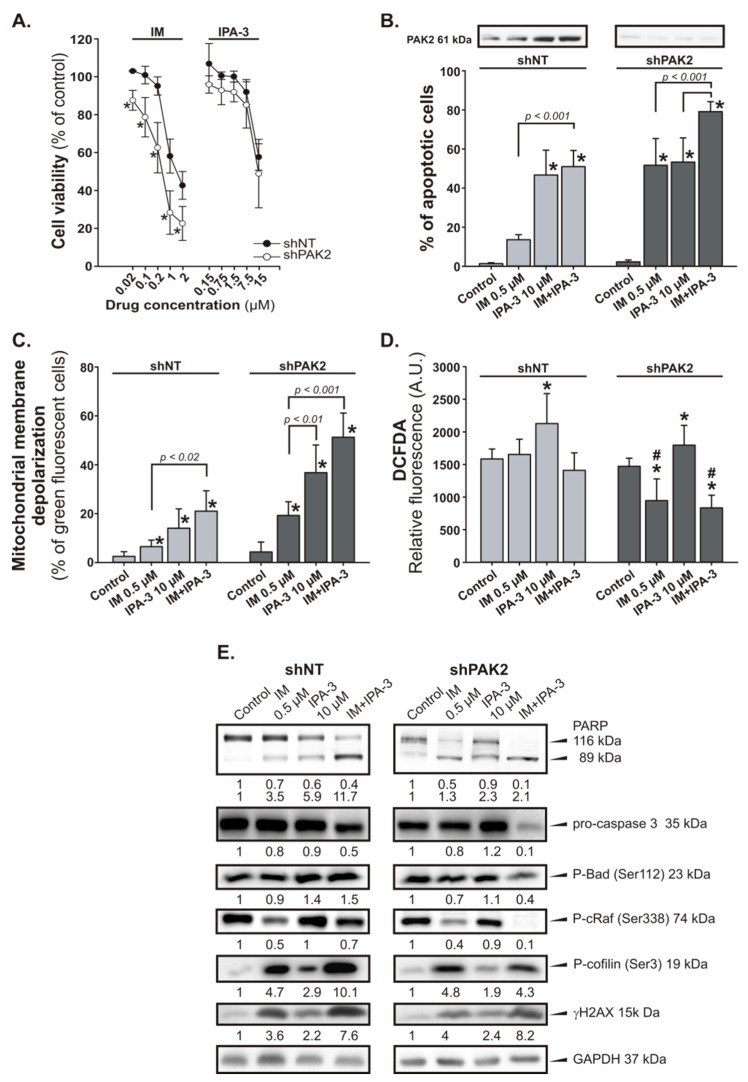
Effect of the combination of IM and IPA-3 on p21-activated protein kinase (PAK)2-silenced 32Dcl^BCR-ABL1^ cells (shPAK2). shPAK2 cells and control (shNT) cells were treated with IM and/or IPA-3 for 24 h as indicated. (**A**) Cell viability was determined using the MTT assay. Each point represents mean ± SD (*n* ≥ 5), * *p* < 0.05 in comparison to untreated cells. (**B**) Apoptosis detection. Data are expressed as the mean annexin V-FITC/PI staining ± SD (*n* = 5). * *p* < 0.001 in comparison to control (**C**). PAK2 expression level in shNT and shPAK2 cells was determined by Western blot analysis. (**C**) Changes in the mitochondrial membrane potential. Green fluorescence of monomeric form of JC-1 was detected, indicating dissipation of Δ*Ψm*. Mean ±  SD (*n* = 4), * *p* < 0.05 value in comparison to (**C**). (**D**) Intracellular levels of reactive oxygen species (ROS) in shPAK2 cells. Data are represented as the mean DCFDA staining ± SD, * *p* ≤ 0.03 when compared to (**C**), # *p* < 0.05 when compared to the corresponding shNT cells. (**E**) Western blot of the indicated proteins; GAPDH was used as a loading control. The changes in the protein levels were estimated by quantification of band intensities with the QuantiScan ver.3.0 (Biosoft, UK) densitometry analysis software.

To specify the potential role of PAK2 in CML cells, we attempted to knock down the expression the *PAK2* gene in 32Dcl^BCR-ABL1^ cells using short hairpin RNA (shRNA) constructs. According to our results, shPAK2 cells were more sensitive to IM than that of control cells (shNT; shRNA with non-targeting sequence) and the observed effect was associated with strong induction of apoptosis (Figure 4A). In addition, the reduction of PAK2 expression level after transfection of cells with a vector carrying PAK2 shRNA construct was confirmed by Western blot analysis (Figure 4B). The treatment of shPAK2 cells with IM induced apoptosis in ~55% of the cells, whereas in the control group, the level of apoptotic cells achieved was ~20% (Figure 4B). It is noteworthy that the combination treatment further increased the level of apoptotic cells (~80%) (Figure 4B). IM and IPA-3-induced apoptosis through the mitochondrial depolarization in shNT and shPAK2 cells (Figure 4C). However, shPAK2 cells were more sensitive to IM and IPA-3. In this case, the percentage of cells with depolarized MMP displayed ~2.5-fold increase in shPAK2 cells in comparison to shNT cells. In addition, the incubation of shPAK2 cells with 0.5 µM IM or its combination with 10 µM IPA-3 significantly reduced the level of ROS (*p* ≤ 0.034) in comparison to control group (Figure 4D). The induction of apoptosis was also confirmed by the decreased level of pro-caspase 3 and a concomitant increase in the cleavage of the PARP in cells treated with combination of IM and IPA-3 (Figure 4E). We also observed changes in the level of phosphorylation of proteins as cRaf at Ser338 and BAD at Ser112, and the response of cells with silenced *PAK2* was more marked (Figure 4E). To verify the effect of IPA-3 on PAK activity, we evaluated the status of phosphorylation of the PAK effector, cofilin at Ser3. We observed an elevated level of phosphorylated cofilin at Ser3 after treatment of cells with IM and IPA-3 (Figure 4E). The most pronounced effect was observed in shNT cells treated with IM alone or in combination with IPA-3. In shPAK2 cells, the observed effect was weaker than that observed in shNT cells especially those treated with the combination. We have also tested the level of DNA damage marker the γH2AX, as the possible indicator of integrity and proper functioning of cells. Western blot analysis revealed that the treatment of cells with IM and IPA-3 stimulates phosphorylation of H2AX protein (Figure 4E).

## 3. Discussion

The effect of group I PAKs and their inhibitors on the development of solid tumors and their possible therapeutic usage has been widely described, whereas their potential role in hematological malignancies has not been extensively explored [40]. Bolton-Gillespie et al. detected elevated levels of phosphorylated PAK in LSCs isolated from patients with CML-CP [35]. Therefore, we hypothesized that PAKs may be a good target to treat CML. In addition, new therapeutic approaches are needed as the patients develop resistance to the existing TKIs, show poor response to the treatment, experience potential toxicity, as well as due to the potential withdrawal of existing TKI therapy in the case of the long-term treated patients [41]. Since PAK1 and PAK2 are expressed in hematopoietic cells and PAK3, the third isoform belonging to group I PAK kinases, is mostly expressed in the brain we focused on PAK1 and PAK2 [42]. Moreover, in silico analysis of the data from Genevesigator platform reveled that PAK1 and PAK2 (but not PAK3, PAK4, PAK5, etc.) are highly expressed in CML indicating a privileged role of PAK1 and/or PAK2 in the pathogenesis of BCR-ABL1 positive diseases [43].

PAK activity has been shown to downregulate several important proapoptotic and signaling pathways and in this context, PAKs may play an important role in the treatment of CML. We found that IPA-3, a small molecule inhibitor of PAK1, can modulate the response of leukemic cancer cells to IM, a first-generation TKI and the gold standard in the treatment of patients with newly diagnosed CML-CP. We have demonstrated the growth inhibition of the leukemic K562 and KCL-22 cells after treatment with IM and IPA-3 alone. The observed effect was comparable to the order of drugs potency calculated on the basis of the percentage of cell survival which increased after combination treatment. The type of interaction between the two drugs was quantitatively analyzed according to the median effect method. The combination index (CI) values of IM and IPA-3 at 50% of the fraction affected (Fa) indicated synergistic (CI < 1) type of interaction between the evaluated agents. Similar effect was seen in the clonogenic assay. The combination treatment of Lin¯CD34^+^ cells derived from patients with CML-CP resulted in stronger suppression of colony formation in comparison to individual treatment. This implies improved efficacy of the combination treatment at this phase of the disease, especially when the concentration of IM in combination therapy is lower than that normally used, at a range of 1–5 μM, in in vitro studies (at 400 mg/day, the current standard dose for CML-CP, peak levels at steady state is ~5 µM) [44].

The inhibitory effect of combination treatment resulted from perturbations in the cell cycle progression and induction of apoptosis [45]. Indeed, IPA-3 effectively induced apoptosis in LSC-enriched CD34^+^38^−^ and LPC-enriched CD34^+^38^+^ cells and in K562 and KCL-22 CML cells most likely via activation of mitochondrial pathway. This might be due to the loss of MMP. In addition, incubation of the cells with IM and the combination resulted in the decrease in the level of pro-caspase 3 and cleavage of PARP protein. Fragmentation of PARP protein indirectly confirms the increased activity of caspases-3 and indicates starting an executive phase of apoptosis.

We hypothesize that PAKs inhibit their downstream effectors, namely, LIM1, 2 serine/threonine kinases (LIMK), which regulate the progression of the cell cycle by prolonged activation of mitogen-activated protein kinase (MAPK) and phosphorylation of cofilin on Serine 3 [46]. The active MAPK may stimulate the proliferation/survival of the cancer cell, whereas the inactivation of cofilin by its phosphorylation, leads to F-actin polymerization, which in turn may cause a decrease in the MMP (*Ψ_m_*) and enhance ROS production. This might also be true in CML-CP stem cell progenitors [47,48]. Therefore, blocking PAK activity may affect the CML cell survival in two ways: (i) by decreasing the activity of LIMK which will result in the reduction of ROS generation especially in CML stem cells and (ii) by induction of apoptosis in CML cells. Our results did not support the former mechanism because cofilin, the direct target of LIMK, remained phosphorylated on Serine 3. It has been shown that other kinases than LIMK are able to phosphorylate cofilin at Serine 3 [49,50]. Elevated levels of phosphorylated cofilin at Serine 3 indicate its higher transition to the nucleus. In the nucleus, phosphorylated cofilin may localize around DNA thereby hampering the recognition of H2A histone family member X (H2AX) by ataxia-telangiectasia mutated (ATM) kinase leading to an insufficient DNA repair leading to cell death [51]. PAK activity has been shown to downregulate several important proapoptotic and signaling pathways and in this context, PAK may play an important role in CML therapy. We found that depending on the cell types, the combination of IM with IPA-3 downregulated the activity of cRaf kinase (decreased level of phosphorylated cRaf at Ser338). Our results seem to be consistent with the present knowledge that phosphorylation of cRaf at Ser338 is dependent on PAK kinase. Low activity of cRaf caused a decreased level of phosphorylation of its downregulated targets such as BAD protein at Ser112 and ERK1/2 kinase (only in KCL-22 cells). BCR-ABL1 kinase may also interact with cRaf to alter the phosphorylation of BAD at the mitochondrial membrane, and in this way, it might prevent the induction of apoptosis in CML cells [52]. Therefore, cells sensitive to IM and IPA-3 present an expected pattern of changes in the level of proteins involved in apoptosis and signaling pathways. This, in some way, confirms that the final effect of the combination treatment may depend on the genetic background of the cells. In our study, K562 cells seemed to be less sensitive to IPA-3 in comparison to KCL-22 cells. Our observation indirectly is consistent with the results obtained by Kuzelowa et al. who showed that K562 cells are relatively resistant to IPA-3-induced cell death [45]. On the other hand, in comparison to shNT cells, IM treated shPAK2 cells appear to be more sensitive to apoptosis and this response increased when PAK1 is additionally inhibited. We also observed the elevated level of PAK2 in K562 cells treated with IPA-3. The results suggested that in some cases ubiquitous PAK2 probably played a compensatory role for the loss of PAK1. Our study showed that inhibition of PAK in CML cell lines, as well as in primary CML-CP cells, may enhance the response to IM treatment. Moreover, because of resistance emerging in patients treated with IM or recurrence of the disease after drug discontinuation, TKIs of new generation are developed. We showed that IPA-3 in combination with dasatinib or ponatinib, the 2nd and 3rd generation TKIs, respectively, could exhibit synergistic or additive types of interaction as indicated by CI values at 50% of the fraction affected (Fa). Obtained results also indicated that such combinations could approach positive kinds of interaction at higher Fa levels (Figure S2). Although we cannot clearly distinguish which isoform, PAK1 and/or PAK2, is responsible for the observed effect, PAK1/2 may be taken into consideration as targets for the treatment of CML.

## 4. Material and Methods

### 4.1. Patient Samples and Cell Culture

The human BCR-ABL1 positive K562 and KCL-22 cell lines were obtained from DSMZ-German Collection of Microorganisms and Cell Cultures and were routinely cultured in RPMI 1640 medium (BioWest, Cytogen, Zgierz, Poland) supplemented with 10% (*v*/*v*) heat-inactivated fetal bovine serum (FBS, BioWest), 2 mM stable-glutamine (BioWest), 100 units/mL penicillin, 100 µg/mL streptomycin, and 250 ng/mL amphoterycin (BioWest). The 32Dcl^BCR-ABL1^ cell line was previously constructed in T. Skorski’s laboratory by transformation of murine 32Dcl3 hematopoietic cells with *p210BCR-ABL1* [53]. The cells were used and maintained in Iscove’s modified defined medium supplemented with 10% (*v*/*v*) heat-inactivated fetal bovine serum (FBS, BioWest), 2 mM stable-glutamine (BioWest), 100 units/mL penicillin, 100 µg/mL streptomycin and 250 ng/mL amphoterycin (BioWest).

Bone marrow samples, at diagnosis from patients with CML-CP and CML-BP (progressed from CML-CP), were obtained from the Department of Hematology, Military Institute of Medicine, Warsaw, Poland. Healthy donor samples were purchased from Cambrex BioScience (Walkersville, MD, USA). Mononuclear cells were obtained by density centrifugation on Histopaque-1077 (Sigma-Aldrich, Poznań, Poland) in SepMate tubes (StemCell Technologies, Vancouver, Canada). Lin¯CD34^+^ cells were separated by magnetic sorting using the EasySep Human Progenitor Cell Enrichment Kit followed by the EasySep™ Human CD34 Positive Selection Kit cocktail (StemCell Technologies). After isolation, the cells were suspended in StemPro-34 serum free medium (SFM) (Gibco, Life Technologies, Warsaw, Poland) in the presence of the following growth factors (PeproTech, London, UK): 100 ng/mL stem cell factor (SCF), 100 ng/mL Fms-related tyrosine kinase 3 (Flt-3) ligand, 20 ng/mL interleukin-3 (IL-3), and 20 ng/mL IL-6 [54]. The study was approved by the Ethical Committee of Military Institute of Medicine (protocol code 11/WIM/2015) and involved 10 patients with newly diagnosed CML as verified by fluorescence in situ hybridization (FISH) of their bone marrow aspirate. All cells types were incubated at 37 °C in a humidified atmosphere with 5% CO_2_.

### 4.2. Drugs

Depending on the type of cell line or type of cells, the following concentrations of the tested compounds were used: K562 and 32Dcl^BCR-ABL1^ (shNT and shPAK2) cells were treated with 0.5 µM IM (Sigma-Aldrich, Poznań, Poland) and 10 µM IPA-3 (Sigma-Aldrich) for 24 h. KCL-22 cells were treated with 1 µM IM and 10 µM IPA-3 for 48 h (two doses of the compounds at time 0 and 24 h later). Patient cells were treated with 0.5 µM IM and 10 µM IPA-3 for 48 h (two doses of the compounds at time 0 and 24 h later). Drugs were dissolved in 100% dimethylsulfoxide (DMSO, Sigma-Aldrich) and then diluted in the media for experiments. The final concentration of DMSO, without affecting cell survival, was maintained at 0.2%.

### 4.3. 3-(4, 5-dimethylthiazol-2-yl)-2,5-diphenyltetrazolium bromine (MTT) Assay

Cells were seeded into 96 well plates at a density of 5 × 10^3^ cells/well. After treatment with IM, IPA-3, or their combination, 20 µL of 3-(4, 5-dimethylthiazol-2-yl)-2,5-diphenyltetrazolium bromine (MTT) at the concentration of 5 mg/mL was added at 24, 48, and 72 h. Then cells were incubated for an additional 4 h at 37 °C, and next 180 µL of solubilization solution (10% SDS) was added. The mixture was incubated at RT overnight. The product of solubilized formazan was spectrophotometrically quantified using a microtiter plate reader, Power Wave XS (Bio-Tek, Winooski, VT, USA), at 570 nm wavelength.

### 4.4. Analysis of Drug Interactions

K562 and KCL-22 cells were simultaneously incubated for 72 h with IM, IPA-3, or their combination. The median-effect method was used to analyze the nature of drug–drug interactions and was based on the determination of the combination index (CI) parameter. Data obtained from the experiments of cytotoxicity were used to calculate the CI values over a range of Fa levels from 0.05 to 0.95 (5–95% cell death). CIs of < 1 indicate synergism, CIs equal to 1 indicate additivity, and CIs > 1 indicate antagonism [55]. Mathematical and quantitative analysis of the data was generated by CalcuSyn version 2.0 software (Biosoft, Cambridge, UK).

### 4.5. Analysis of Cell Cycle

Cells (~1.5 × 10^5^) were incubated at −20 °C for 24 h after fixation in ice-cold 80% ethanol. Before staining, cells were washed twice in phosphate-buffered saline (PBS) and then stained with propidium iodide (PI)/RNase solution (50 µg/mL PI, 100 µg/mL RNase in 0.1% PBS/Triton X-100 (PBST) solution) for 30 min in the dark at 4 °C. The samples were then measured using a CyFlow Cube 8 (Sysmex, Norderstedt, Germany) or BD FACSCanto II (BD Biosciences, San Jose, CA, USA) flow cytometer. The DNA histograms were analyzed using FCSExpress 5Flow software (De Novo Software, Glendale, CA, USA).

### 4.6. Analysis of Apoptosis

Apoptosis was measured using annexin-V/ fluorescein isothiocyanate (FITC) (BD Pharmingen, San Jose, CA, USA). The staining procedure was conducted in accordance with the manufacturer’s instructions. The cells were harvested after treatment, washed twice with PBS, and then centrifuged. The cell pellet was resuspended in ice-cold binding buffer and annexin-V/FITC was added to the cell suspension. After 15 min of incubation in the dark at room temperature, the samples were analyzed using a flow cytometer.

### 4.7. ROS Analysis

Cells were incubated with redox-sensitive fluorochrome 2’,9’-dichlorodihydrofluorescein-diacetate (DCFDA) (Sigma-Aldrich) and analyzed using the CyFlow Cube 8 or FACSCanto II flow cytometry systems. DCFDA detects H_2_O_2_ and •OH.

### 4.8. Oxidative DNA Damage

To detect 8-oxoG, cells were stained with the DNA/RNA damage antibody-FITC conjugated (StressMarq Biosciences Inc., Victoria, Canada) to assay for DNA double-strand breaks (DSBs) by flow cytometry. Briefly, cells were fixed with 1% paraformaldehyde on ice for 15 min. After washing in PBS, the cells were permeabilized in 0.1% PBST for 30 min at room temperature and then washed again in 0.01% PBST (230 × g for 5 min). Next, 100 μL of the diluted FITC conjugated antibody was added to the cell pellet and incubated for 1 h at RT in the dark. Cells were washed again in the washing solution (230 × g, 10 min), and the pellet was resuspended in PBS and subsequently measured with a flow cytometer.

### 4.9. Immunoblotting

The cells were washed with cold PBS buffer and total proteins were extracted from the collected cells using RIPA buffer (25 mM Tris–HCl (pH 7.4), 150 mM NaCl, 0.1% SDS, 0.5% sodium deoxycholate, 1mM EDTA, 1% Nonidet P-40) and protease inhibitors. The bicinchoninic acid (BCA) assay was performed to determine the total protein concentration in the samples (Pierce, Rockford, IL, USA). Samples were fractionated on a 4–20% SDS-PAGE gels. Next, the proteins were transferred onto a nitrocellulose membrane and probed with anti-human/mouse antibodies specific to PARP (#9542), caspase 3 (#9668), p-BAD Ser112 (#9291), p-cRaf Ser338 (#9427), p-ERK1/2 Thr202/Tyr204(#4370), p-PAK1 Thr423/PAK2 Thr402 (#2601), PAK2 (2608), p-cofilin Ser3 (#3313), γH2AX (#9718), p-STAT3 Tyr705 (#9138), and GAPDH (#5174) (purchased from Cell Signaling Technologies, LabJOT, Warsaw, Poland). Signals were detected using the enhanced chemiluminescence detection system (MicroChemi Bio-Imaging Systems). The images of row Western blot membranes are shown in Figure S2.

### 4.10. Measurement of Mitochondrial Membrane Potential (ΔΨ_m_)

The *ΔΨ_m_* was measured by flow cytometry using MitoStatus Red (BD Pharmingen), a cationic lipophilic dye which accumulates within the mitochondria of healthy cells, or by using JC-1 (5,5′,6,6′-tetrachloro-1,1′,3,3′-tetraethylbenzimidazolo-carbocyanine iodide), a dye which remains in monomeric form in the cytoplasm of apoptotic cells and emits green fluorescence. Cells were stained according to the manufacturer’s instructions and examined by flow cytometer.

### 4.11. Colony-Forming Assay

For in vitro colony-forming test, 2 × 10^3^ CD34^+^ cells were plated in triplicate in StemPro-34 medium and growth factors as described earlier. IM, IPA-3, and their combination were added for 72 h followed by plating in methylcellulose-based medium MethoCult H4230 (StemCell Technologies) in the presence of growth factors. Colonies were counted after 7–10 days using an inverted microscope.

### 4.12. shRNA Transfection

PAK2 mouse shRNA pRFP-C-RS vectors (TF506650, OriGene, MD, USA), were transfected into 32Dcl^BCR-ABL1^ cells using electroporation system GenePulser Xcell (Bio-Rad, Hercules, CA, USA). Cells expressing RFP fluorescent protein were separated and collected by the use of the FACS Aria (BD Biosciences). In addition, cells were selected and maintained in media containing 2 µg/mL puromycin.

### 4.13. Statistical Analysis

Data are presented as mean values ± standard deviation (SD). Statistical comparisons among groups were performed by Student’s *t*-test or one-way analysis of variance (ANOVA) followed by Student–Newman–Keuls post-hoc test. Significance was assumed at *p* < 0.05 (marked with asterisks or crosses on graphs).

## 5. Conclusions

In conclusion, our results support our opinion that PAKs may be taken into consideration as the possible target in CML treatment. Although the potential benefits of using PAK inhibitors in CML treatment needs further evaluation, the search for new, more specific PAK inhibitors seem to be rational and suggest a potential therapeutic application of PAK inhibitors.

## Supplementary Materials

The following are available online at https://www.mdpi.com/2072-6694/11/10/1544/s1, Figure S1: The level of pCrkl in K562 and KCL-22 cells treated by imatinib (IM) or/and IPA-3, respectively, Figure S2: Effect of dasatinib (DAS), ponatinib (PON) and IPA-3 on cell viability of CML-BP cell lines, Figure S3: Row Western blots for Figure 2 and Figure 4.
