# Supplementary material for: Simultaneous Inhibition of BCR-ABL1 Tyrosine Kinase and PAK1/2 Serine/Threonine Kinase Exerts Synergistic Effect against Chronic Myeloid Leukemia Cells
Source: Cancers (Basel). 2019 Oct 12;11(10):1544. doi: 10.3390/cancers11101544 (PMC6826736; doi:10.3390/cancers11101544)
Supplement: Supplementary file 1 [file cancers-11-01544-s001.zip › cancers-574215-SI/cancers-574215 supplementary final/cancers-574215 figure S1.pdf]

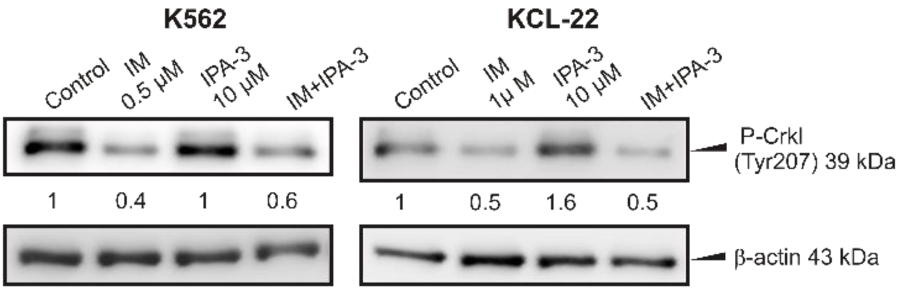

Figure S1. The level of pCrkl in K562 and KCL-22 cells treated by imatinib (IM) or/and IPA-3, respectively. Western blots of total lysates, β-actin was used as a loading control.
