# Supplementary material for: Simultaneous Inhibition of BCR-ABL1 Tyrosine Kinase and PAK1/2 Serine/Threonine Kinase Exerts Synergistic Effect against Chronic Myeloid Leukemia Cells
Source: Cancers (Basel). 2019 Oct 12;11(10):1544. doi: 10.3390/cancers11101544 (PMC6826736; doi:10.3390/cancers11101544)
Supplement: Supplementary file 1 [file cancers-11-01544-s001.zip › cancers-574215-SI/cancers-574215 supplementary final/cancers-574215 figure S2.pdf]

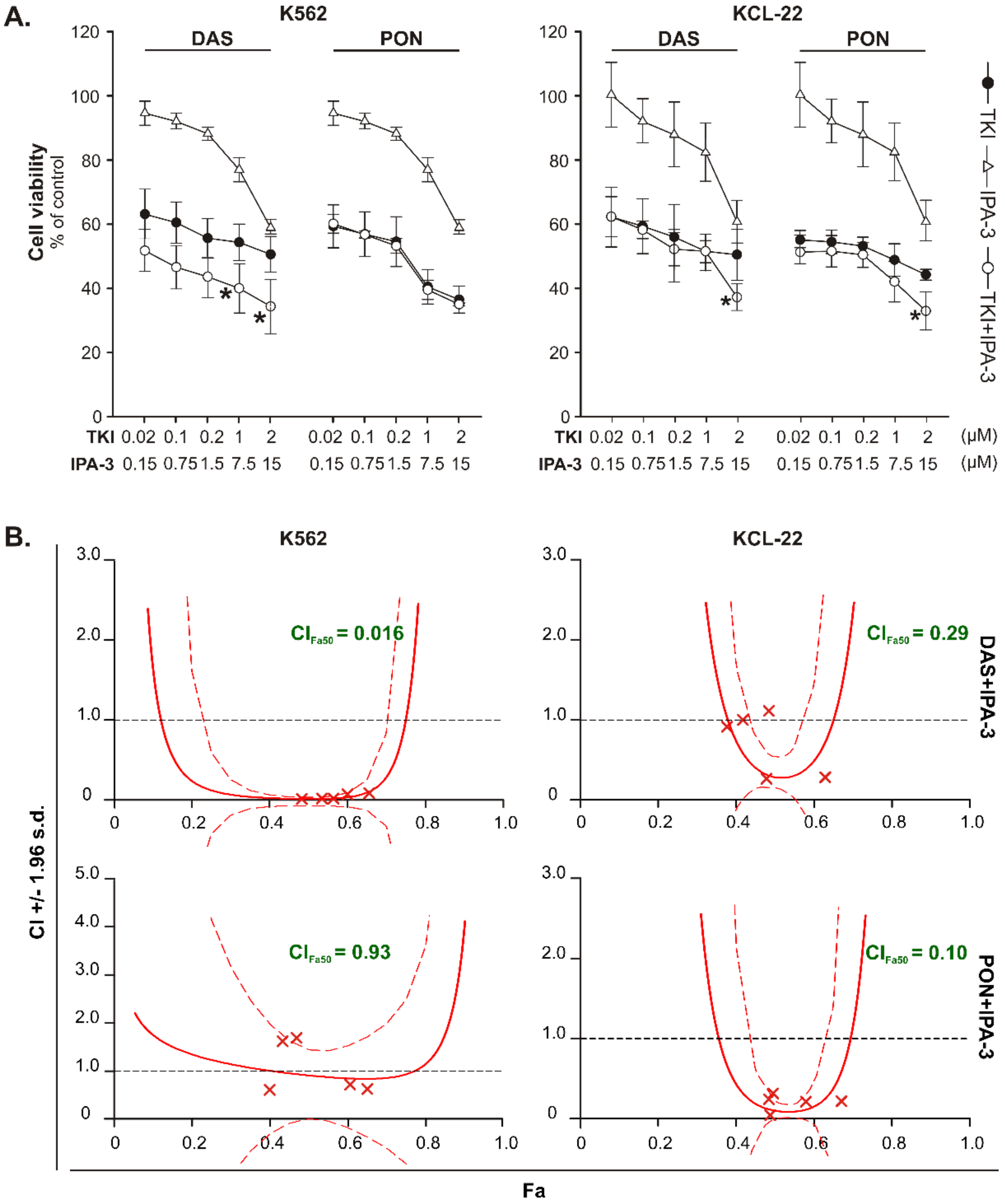

Figure S2. Effect of dasatinib (DAS), ponatinib (PON) and IPA-3 on cell viability of CML-BP cell lines. (**A**) The cells were treated for 24 h with the indicated doses of TKIs and IPA-3 individually and in combination followed by MTT assay to test cell viability. Each point represents the mean ± standard deviation (SD) ($n \geq 4$), * $p < 0.05$ in comparison with TKI. (B) Combination index (CI) values with a 95% confidence interval at all effective levels indicate the nature of the interactions (CI =1 = additivity, CI <1 = synergy, CI >1 = antagonism). Fa is the fraction of cells affected by combination treatment.
