# Supplementary material for: Simultaneous Inhibition of BCR-ABL1 Tyrosine Kinase and PAK1/2 Serine/Threonine Kinase Exerts Synergistic Effect against Chronic Myeloid Leukemia Cells
Source: Cancers (Basel). 2019 Oct 12;11(10):1544. doi: 10.3390/cancers11101544 (PMC6826736; doi:10.3390/cancers11101544)
Supplement: Supplementary file 1 [file cancers-11-01544-s001.zip › cancers-574215-SI/cancers-574215 supplementary final/cancers-574215 figure S3.pdf]

Figure S3. Row Western blots for Figures 2 and 4. The order of the samples are the same as on the Figures 2 and 4.

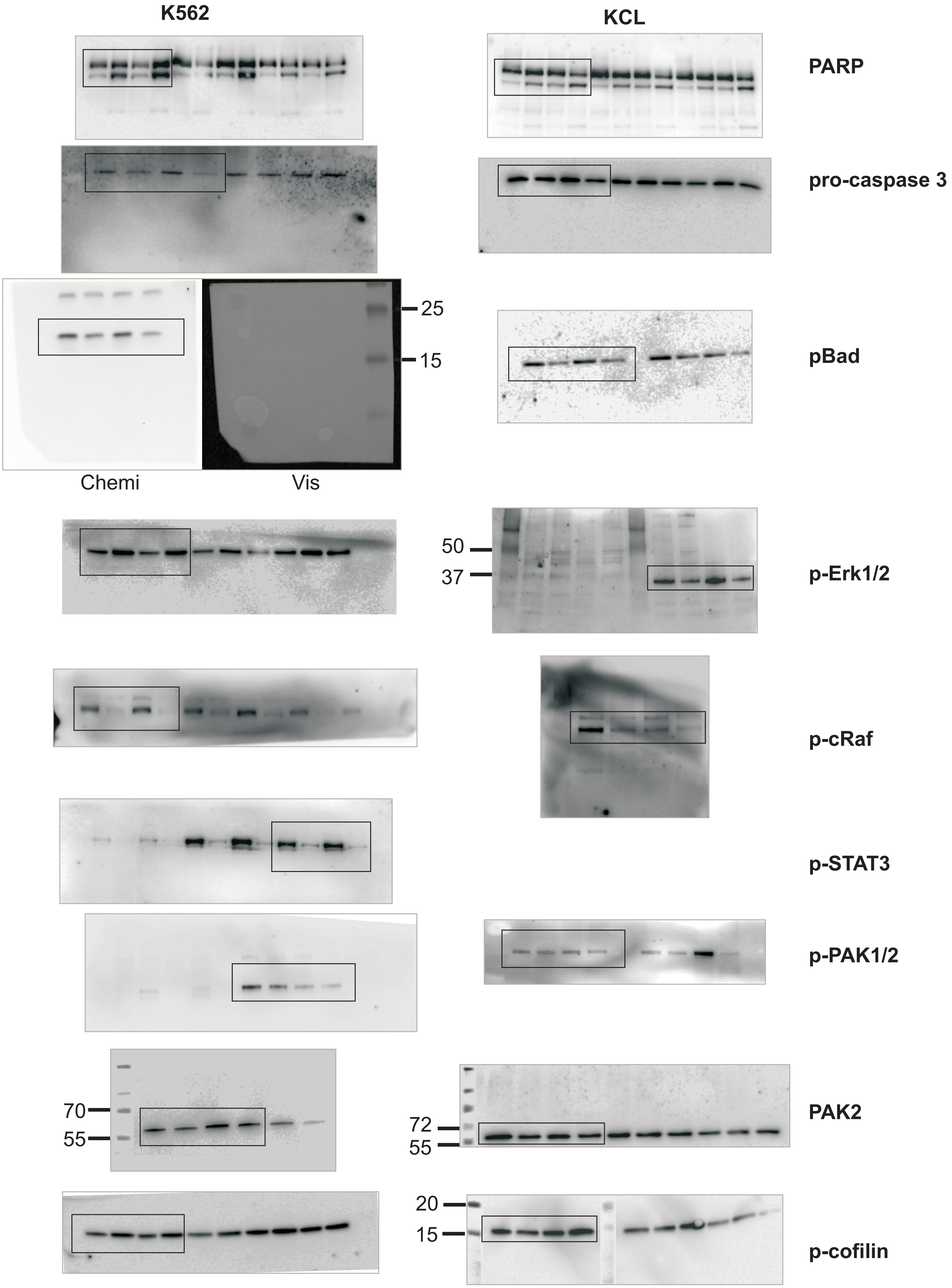

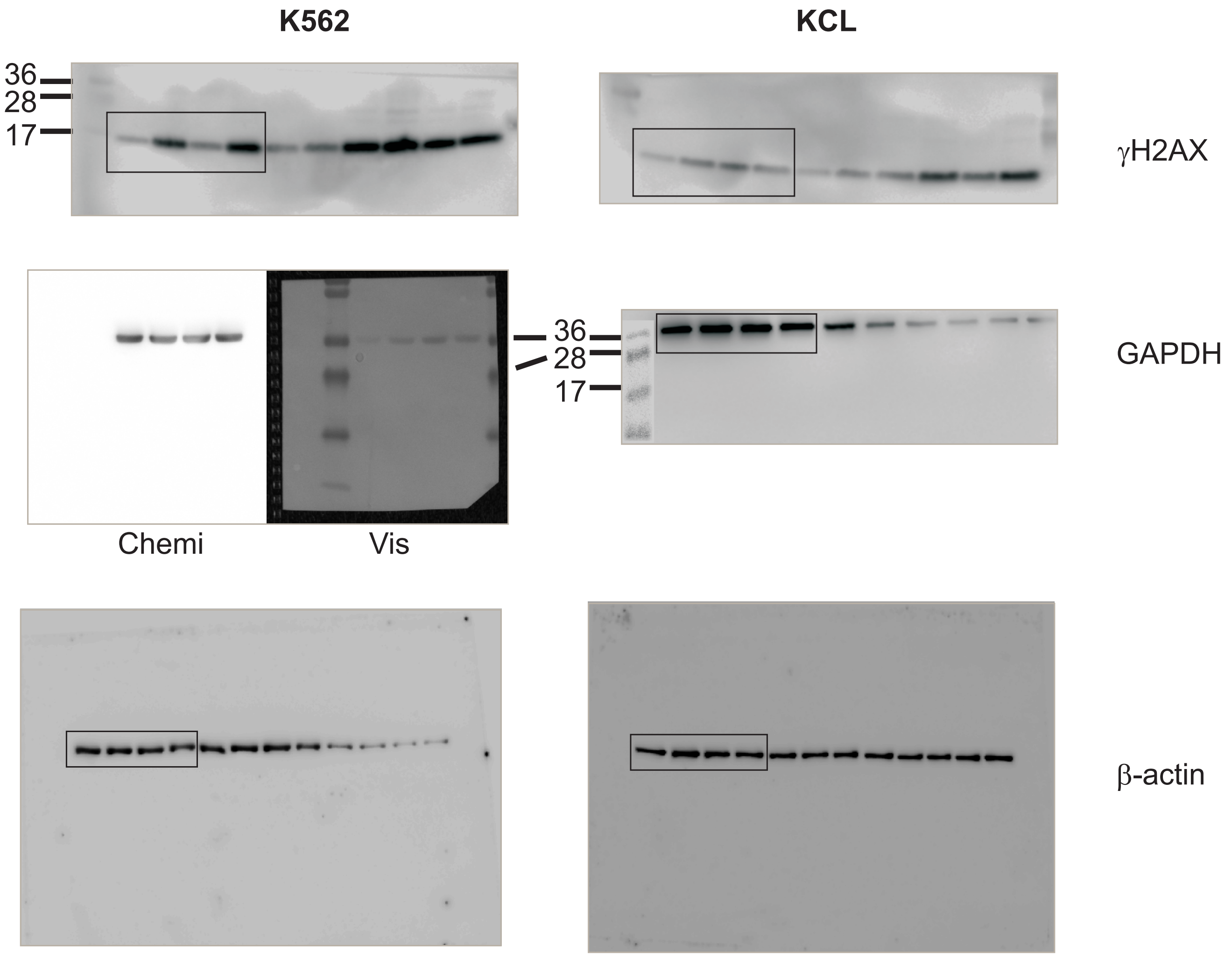
K562
KCL
36
28
17
γH2AX
36
28
17
GAPDH
Chemi
Vis
β-actin

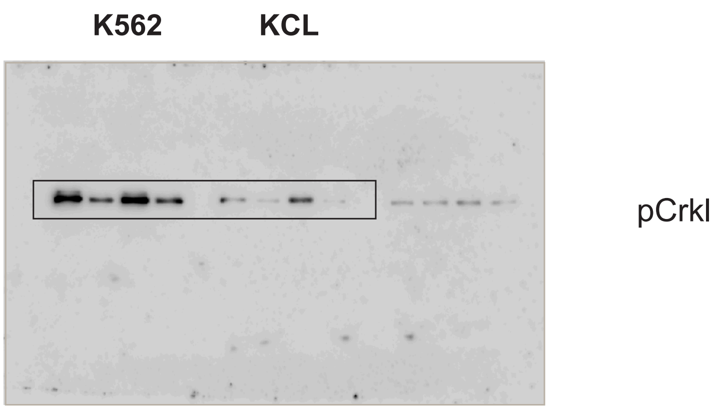
K562
KCL
pCrkl

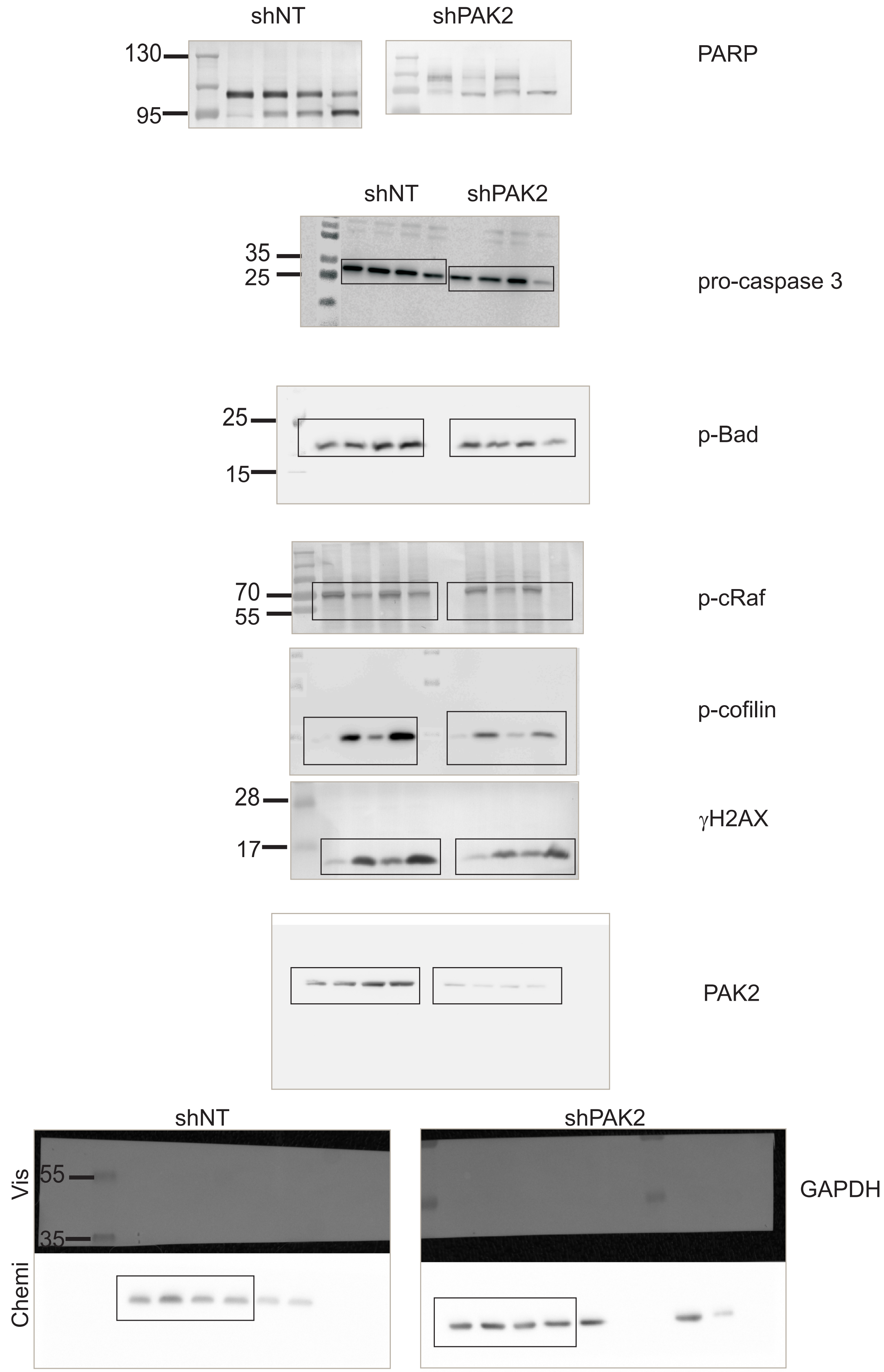
shNT
shPAK2
130
95
PARP
shNT
shPAK2
35
25
pro-caspase 3
25
15
p-Bad
70
55
p-cRaf
p-cofilin
28
17
γH2AX
PAK2
shNT
shPAK2
Vis
55
35
Chemi
GAPDH
